# Spatiotemporal Infectious Disease Modeling: A BME-SIR Approach

**DOI:** 10.1371/journal.pone.0072168

**Published:** 2013-09-27

**Authors:** Jose Angulo, Hwa-Lung Yu, Andrea Langousis, Alexander Kolovos, Jinfeng Wang, Ana Esther Madrid, George Christakos

**Affiliations:** 1 Department of Statistics and Operations Research, University of Granada, Granada, Spain; 2 Department of Bioenvironmental Systems Engineering, National Taiwan University, Taipei, Taiwan; 3 Department of Civil Engineering, University of Patras, Patras, Greece; 4 SpaceTimeWorks, LLC, San Diego, California, United States of America; 5 Institute of Geographic Sciences & Natural Resources Research, Chinese Academy of Sciences, Beijing, China; 6 University Centre of Defence at the Spanish Air Force Academy, MDE-UPCT, Santiago de la Ribera, Murcia, Spain; 7 Department of Geography, San Diego State University, San Diego, California, United States of America; 8 Department of Environment & Natural Resources, Zhejiang University, Hangzhou, China; National Institute for Public Health and the Environment, Netherlands

## Abstract

This paper is concerned with the modeling of infectious disease spread in a composite space-time domain under conditions of uncertainty. We focus on stochastic modeling that accounts for basic mechanisms of disease distribution and multi-sourced in situ uncertainties. Starting from the general formulation of population migration dynamics and the specification of transmission and recovery rates, the model studies the functional formulation of the evolution of the fractions of susceptible-infected-recovered individuals. The suggested approach is capable of: a) modeling population dynamics within and across localities, b) integrating the disease representation (i.e. susceptible-infected-recovered individuals) with observation time series at different geographical locations and other sources of information (e.g. hard and soft data, empirical relationships, secondary information), and c) generating predictions of disease spread and associated parameters in real time, while considering model and observation uncertainties. Key aspects of the proposed approach are illustrated by means of simulations (i.e. synthetic studies), and a real-world application using hand-foot-mouth disease (HFMD) data from China.

## Introduction

Understanding infectious disease patterns (i.e. space-time variations and/or changes) has always been a challenging affair. Disease diffusion can vary significantly from place to place and from time to time for a number of reasons, including heterogeneity of the hosts and pathogens, physical and social environments, and interactions across space and time. Moreover, uncertainties linked to population movement and records of infected individuals can increase the difficulty of understanding the spatiotemporal spread of an infectious disease. A number of key studies have shown that infectious disease spread depends significantly upon the spatial features of a population [1]–[5] whereas major benefits of spatial disease modeling include the assessment of disease intervention and control strategies (e.g., border control and quarantine). Accordingly, several models have been proposed to quantify the spatial disease features at both population and individual scales [1], [6]–[8]. Among the best-known models are the gravity, the spatial micro-simulation, and the network models [1], [6], [9]. Most of these models focus primarily on interactions between the susceptible and infected populations across geographical locations, without considering the continuous local population dynamics of disease evolution. This is especially the case for the gravity model, where the geographical distribution and interaction patterns of populations are discretized into separated locations. Stochastic “Susceptible-Infected-Recovered” models (SIR, [10]–[13]) have been widely implemented to represent disease evolution of populations over time. Spatial metapopulation approaches extend SIR models to explicitly account for the local or global population movements between different geographical locations, in terms of patches or networks with deterministic or stochastic characteristics [14]–[16].

The present study proposes a realistic *space-time* extension of a purely temporal SIR model, i.e. metapopulation model, in the context of Bayesian maximum entropy (BME) theory [17], [18]. The space-time BME-SIR model has certain attractive features: (1) it represents the population dynamics of infectious diseases within and across localities; (2) it takes into consideration the composite space-time variation of disease features; (3) it accounts for observation uncertainties (e.g., in the records of infected individuals); (4) in addition to the susceptible-infected-recovered disease dynamics, it integrates different sources of knowledge (e.g., hard and soft disease data together with epidemic models and physical laws); and (5) it updates the space-time model parameters in real time.

## Results

### Theoretical SIR model vs. simulated data

**Figure 1:**
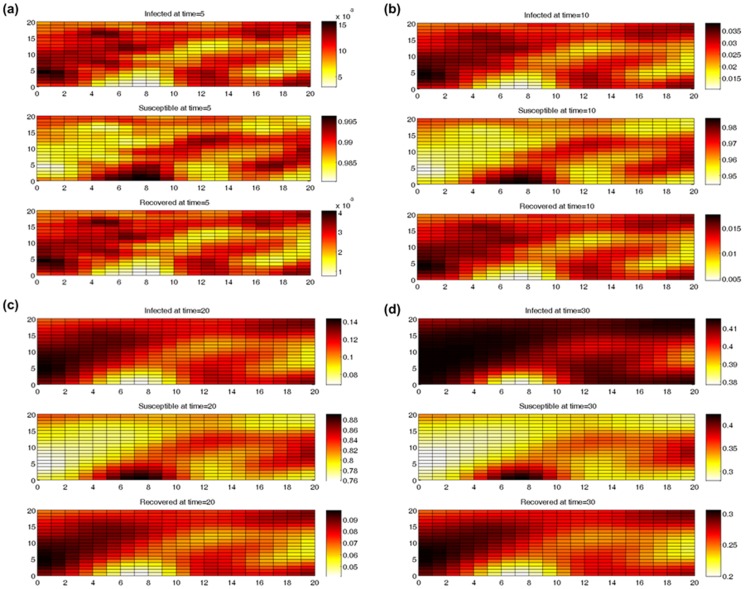
Spatial distribution of SIR population fractions at different times: (a) *t* = 5, (b) *t* = 10, (c) *t* = 20, and (d) *t* = 30.

This simulation (synthetic) study assumes an initial distribution of infected population fraction, 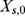, in a gridded domain of size 20×20 square cells with unit area. Subsequently, the space-time distributions of 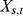, 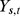, and 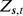 are generated in terms of Monte-Carlo simulation using the SIR model in Eqs. (1a–c). In this study, the sptial variability of 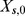 is described by a covariance function of the exponential form 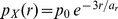, where *r* denotes physical distance, 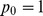 and 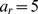. To describe population movement, we used a Gaussian kernel function, 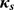, with bandwidth 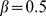. In this simulation, the population portion that resides at a certain location and does not migrate is estimated as
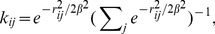
(7)where 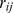 is the distance between grid points *i* and *j*. Equation (7) gives 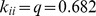, i.e. approximately 70% of the population at each unit cell of the grid has residence time equal to the time step of the simulation. In this case, the recovery and transmission rates are assumed to be 0.1 and 0.4, respectively. The simulated space-time distributions of the infected, 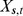, susceptible, 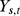, and recovered, 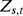, population fractions are plotted in Figures 1a–d for *t* = 5, 10, 20 and 30 (note the changing color scales).

**Figure 2:**
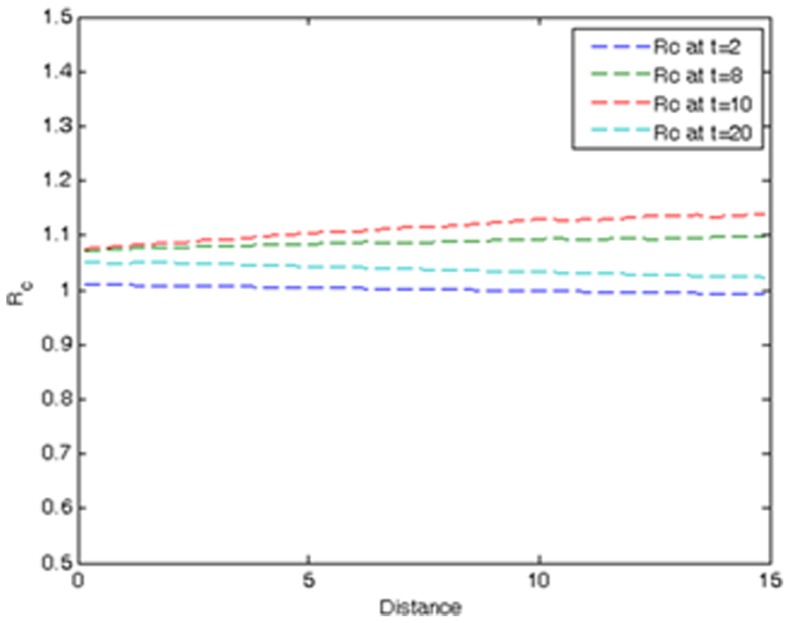
Expected value of the ratio 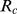
 at times 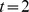, 8, 10 and 20.

Figure 2 shows how the expected value of the ratio 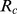 between the right- and left-handsides of Eq. (S2) (see File S1) varies with the distance 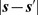 at different times *t* = 2, 8, 10 and 30. One sees that for all times *t* and distances 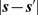, 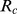 is close to 1 and, hence, the constraint in Eq. (S2) is satisfied to a good approximation. That said, the SIR model in Eqs. (1) can be accurately described using a spatially homogeneous 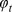-function.

Similar results have also been obtained for different values of the parameters 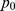, 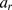 and 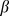 in the corresponding space-time disease covariance models [19]. As noted earlier, 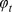 is a monotonically decreasing function of time *t*. In addition, 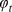 should include at least two parameters that allow variation at 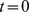 and produce different rates of decay as *t* increases; and a third parameter so that at 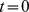, 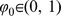, and at 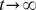, 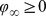. For example, one may choose the function 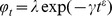, where the parameters 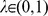 and 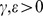. Other possibilities also exist.

**Figure 3:**
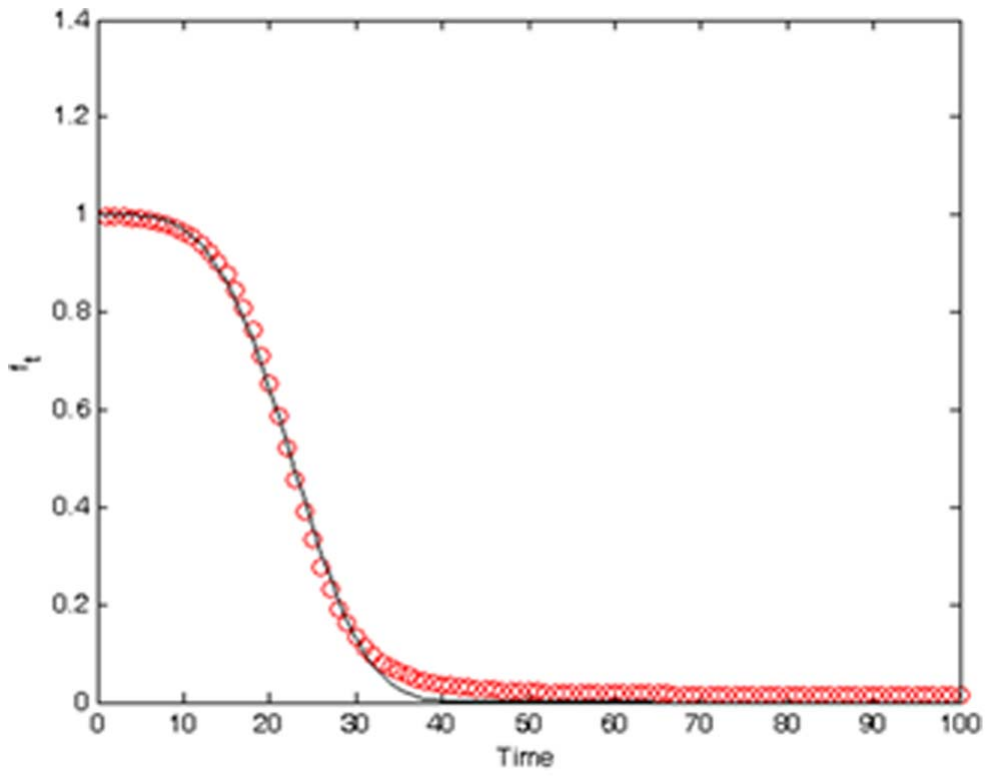
Empirical estimates of the *φ*-function fitted by the functional form 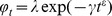
 using the method of least squares.

**Figure 4:**
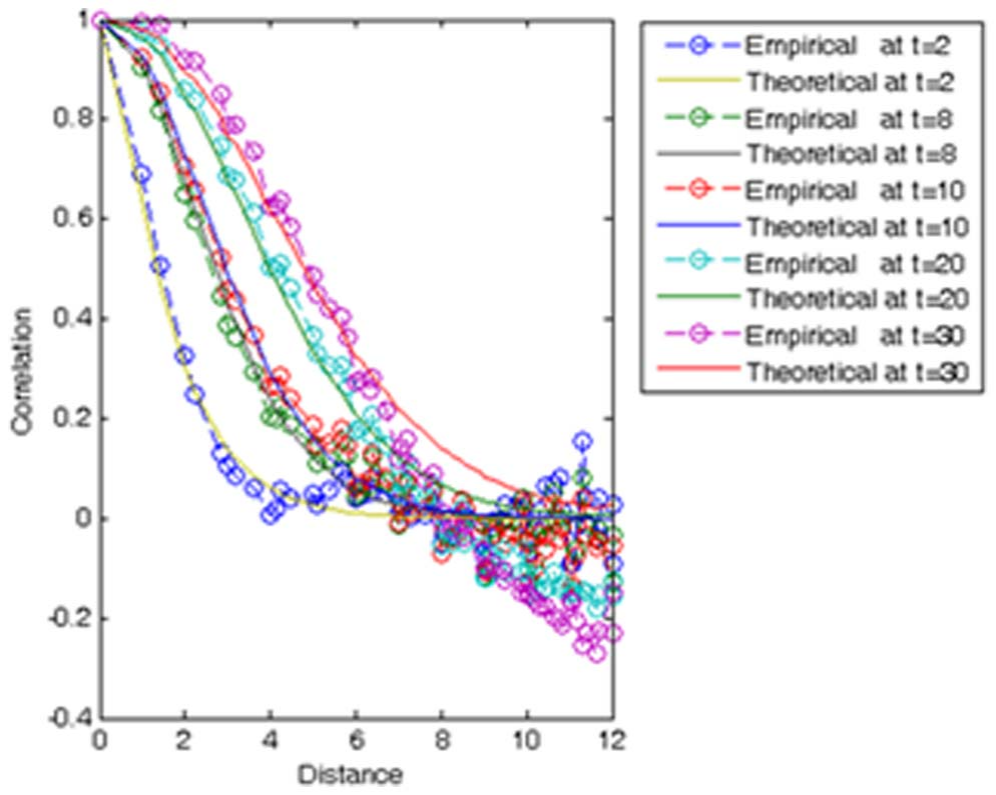
Theoretical and numerically simulated spatial correlation functions of the SIR model for different times; *t* = 2, 8, 10, 20 and 30.

Figure 3 shows empirical estimates of the function 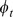 calculated in terms of 300 synthetic realizations of the SIR model in Eqs. (1a–c). One sees that the obtained estimates are fitted well by the functional form 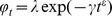 with parameters 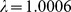, 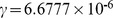, and 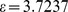. The theoretical covariance of the infected population fraction is calculated from Eq. (S5) (see File S1) using 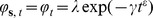 with 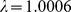, 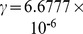, and 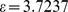. Figure 4 compares the theoretical spatial correlation function of the SIR model at times *t* = 2, 8, 10, 20, and 30 and the associated empirical correlations calculated directly from the simulated infected distributions. Calculation of the former is based on the theoretical covariances obtained from Eq. (S5) (see File S1) using the aforementioned exponential form of the *φ*-function. The latter are empirical estimates of the normalized to max-1 covariance function at different times *t*, obtained through Monte Carlo simulation. For small times *t*<10, one observes a very good fit between the empirical and theoretical covariances across space. However, as *t* increases and for large distances 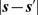 the deviations between simulated and theoretical covariances become larger. We have investigated the matter to some extent and found that this is due to tiny differences in the initial condition 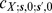, which propagate over time.

### SIR model sensitivity analysis

Next, simulations of the infected (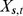), susceptible (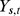) and recovered (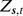) population fractions are generated assuming several numerical values for the SIR parameters. Significant features of the respective temporal evolution curves are illustrated and discussed in relation to the different scenarios.

**Figure 5:**
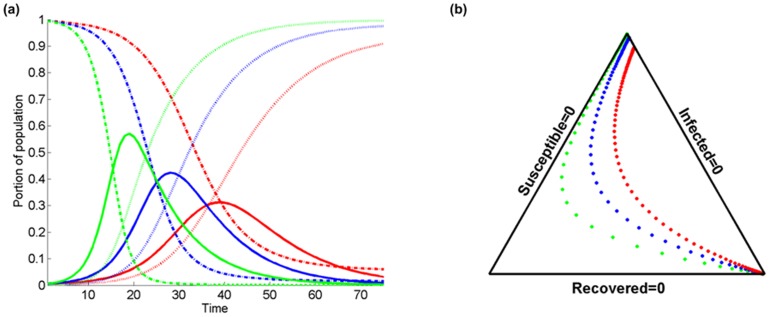
Comparison of the temporal evolution of infected 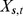 (solid lines), susceptible 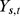 (dashed lines), and recovered 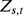 (dotted lines) population fractions at a certain location in terms of (a) the temporal variation with different values of the probability of infection transmission 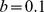 (red color), 0.2 (blue), 0.3 (green). The probability of recovery is 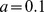, the population fraction that resides inside the domain of interest is 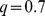, and the kernel bandwidth is 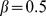, and (b) the simplex triangle plot.

Figure 5a presents a comparison of the temporal evolution of 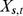 (solid lines), 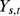 (dashed lines), and 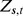 (pointed lines) at a certain location assuming different values of the probability of infection transmission 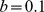 (red color), 0.2 (blue), 0.3 (green); the probability of recovery is 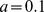, the population fraction that resides at the domain of interest is 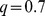, and the kernel bandwidth is 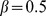. In Fig. 5b, a synthetic representation is given on the simplex triangle 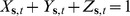 (for a technical discussion, see [20], [21]. The distance between dots corresponds to time intervals starting from the low right corner of the simplex triangle. Several intuitive results are quantitatively represented in Fig. 5a–b. Note that in Fig. 5a, for higher *b* values: (i) the maximum infected fraction is greater and it is reached at an earlier stage; (ii) accordingly, the reduction of the susceptible fraction is faster with time, and (iii) the increase of the recovered population fraction is faster. Moreover, as *b* increases, the limit over time of the susceptible population fraction, that is the population fraction which finally remains unaffected by the disease, tends to be closer to zero. This limiting behavior is more clearly visualized in Fig. 5b.

**Figure 6:**
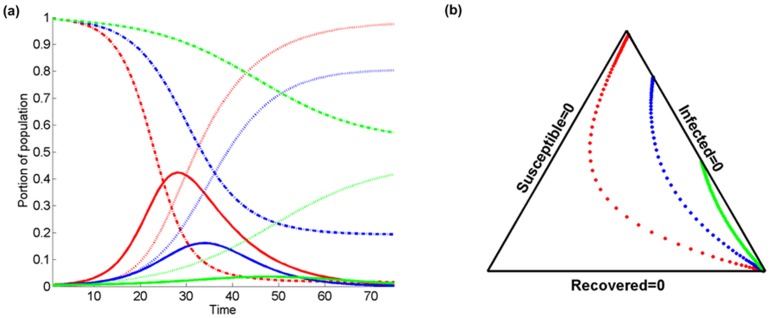
Comparison of the temporal evolution of infected 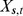 (solid lines), susceptible 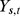 (dashed lines), and recovered 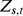 (dotted lines) population fractions at a certain location in terms of (a) the temporal variation with different values of the probability of recovery 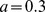 (red color), 0.4 (blue), 0.6 (green). The probability of transmission is 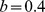, the population fraction that resides inside the domain of interest is 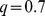, and the kernel bandwidth is 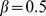, and (b) the associated simplex triangle plot.

Fig. 6a presents a comparison of the temporal evolution of 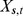 (solid lines), 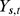 (dashed lines), and 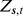 (pointed lines) assuming different values of 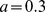 (red color), 0.4 (blue), 0.6 (green). The probability of infection transmission is 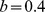, 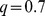, and 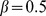. As before, Fig. 6b is a simplex triangle representation of Fig. 6a. Note that variation of *a* leads to the “inverse” SIR behavior than that of *b* in Fig. 5a–b. In Fig. 6a, for smaller values of *a*: (i) the maximum infected fraction is greater and it is reached at an earlier stage; (ii) the reduction of the susceptible fraction is faster with time, and (iii) the increase of the recovered population fraction is faster. In this case, the maximum (over time) of the susceptible population fraction tends to be closer to zero for smaller values of *a*. Note that for *a* = 0.6, more than half of the population remains free of the disease.

**Figure 7:**
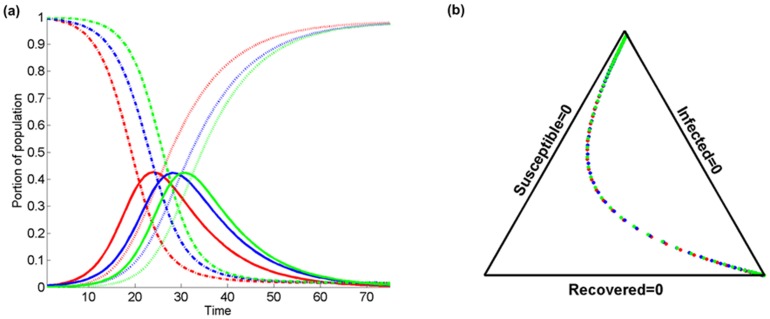
Comparison of the temporal evolution of infected 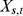 (solid lines), susceptible 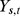 (dashed lines), and recovered 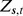 (dotted lines) population fractions at a certain location in terms of (a) the temporal variation of purely temporal model (red color), and the spatiotemporal model for two different kernel bandwidth values 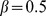 (blue), 3 (green). The probability of recovery is set to 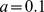; the probability of transmission is 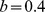, and the population fraction that resides inside the domain of interest is 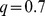, and (b) the associated simplex triangle plot.

Figure 7a presents a comparison of the temporal evolution of 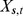 (solid lines), 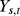 (dashed lines), and 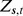 (pointed lines) at a certain location considering the purely temporal model (red color), and the spatiotemporal model for two different kernel bandwidths 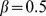 (blue) and 3 (green); with 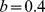, 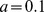, and 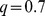. As before, Figure 7b is a simplex triangle representation of Figure 7a. It is worth noting that independently of the value of *β* near all population becomes infected and the maximum infected fraction remains unaffected, although it is reached at an earlier stage for smaller spatial spreads (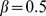 vs. 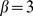). Also, the reduction of the susceptible population fraction is slower with time for larger values of *β* (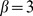 vs. 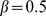). The simplex triangle paths are similar for the three cases, but the SIR velocities are different as reflected in the corresponding inter-point distances.

**Figure 8:**
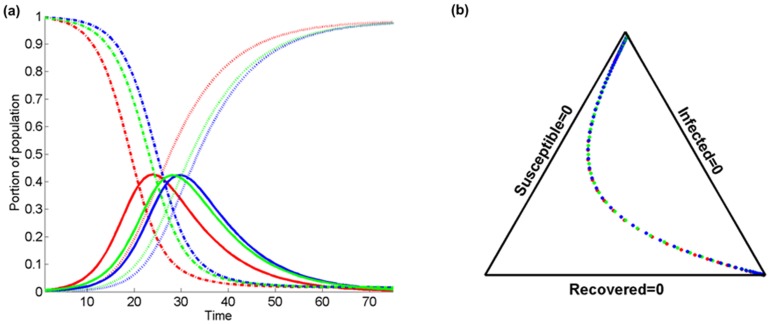
Comparison of the temporal evolution of infected 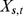 (solid lines), susceptible 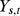 (dashed lines), and recovered 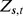 (dotted lines) population fractions at a certain location in terms of (a) the temporal variation of the purely temporal model (red color), and the spatiotemporal model for two different values of the population fraction residing inside the domain of interest 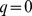 (blue), 0.7 (green). The kernel bandwidth is set to 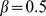, the probability of recovery is 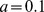, and the probability of transmission is set to 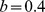. (b) The associated simplex triangle plot.

Figure 8a presents a comparison of the temporal evolution of 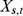 (solid lines), 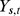 (dashed lines), and 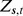 (pointed lines) at a certain location, again considering the purely temporal model (red color), and the spatiotemporal model for two different values of 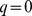 (blue), and 0.7 (green). The kernel bandwidth has been set to 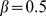, 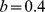, and 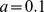. As before, Figure 8b is a simplex triangle representation of Fig. 8a. Note that in Fig. 8a, for 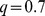 the increase of the infected population fraction (or equivalently the reduction of the susceptible fraction) is faster with time than for 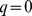. In addition, the maximum infected fraction remains the same, but it is reached faster in the purely temporal case (red color), and when a considerable fraction of the population resides within the domain of interest (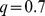 vs. *q* = 0). Similar conclusions can be drawn from the study of the plots in Fig. 8b, with shape similarities translated into coincidental paths with different SIR velocities in the simplex domain.

### A Study of Hand-Foot-Mouth Disease Data

In what follows, the theoretical space-time BME-SIR method is applied to a real-world study of the spread of hand-foot-mouth disease in China (HFMD; [22]). HFMD is the most common infectious disease in China [23], hence there is considerable interest in understanding the evolution of its spatiotemporal patterns and potential correlations to environmental factors. For example, Wang (2011) explores HFMD and climate associations across Eastern China [23]. The HFMD data was obtained from China Center of Disease Control (see File S1).

**Figure 9:**
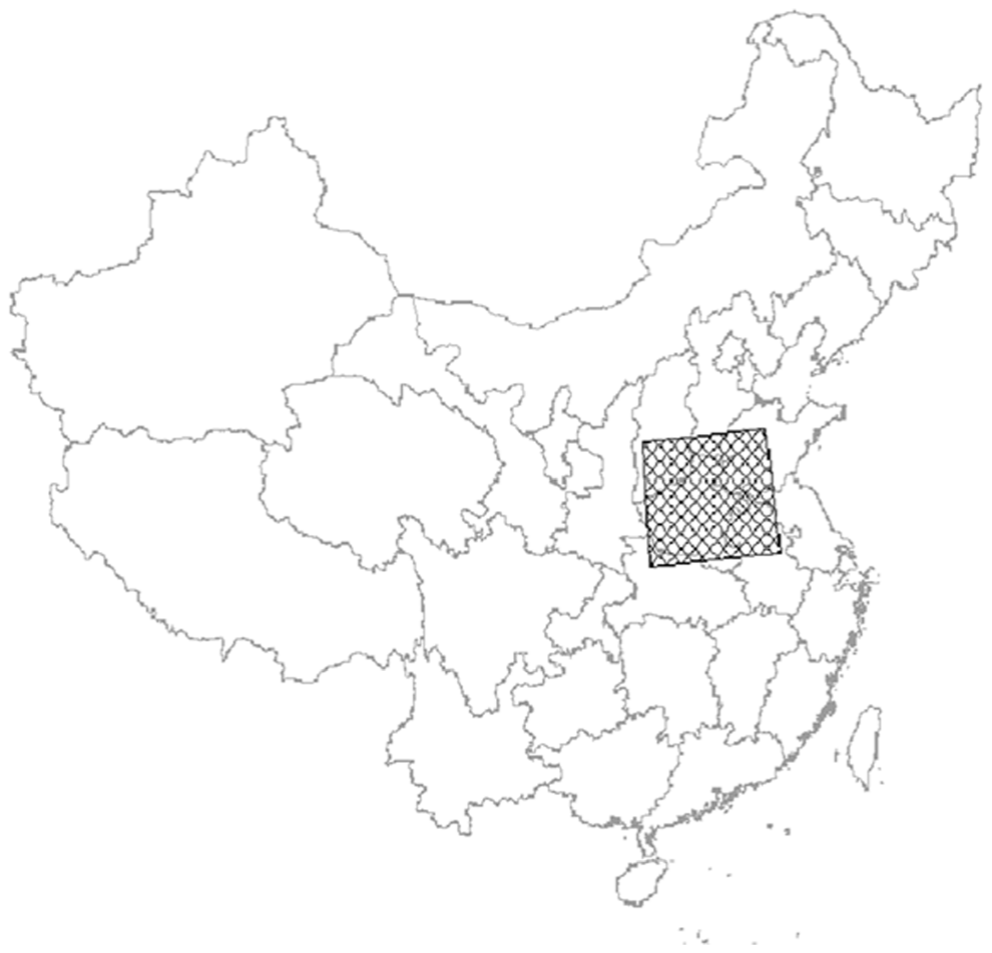
The study region and its location in China.

The study focuses on a specific set of Chinese counties with relatively higher disease incidence; in particular, we focus on the disease evolution in 145 counties that extend between 111°E to 118°E, and 32°N to 37°N (Fig. 9). The data are weekly-aggregated HFMD rates (cases of infecteds per 10000 people) over a period of 20 weeks that span from September 27–October 3, 2008 (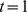) to February 7–13, 2009 (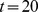). In the example, we account for uncertainty in the data survey by assuming all observations to be uncertain measurements. We consider each rate as a randomly sampled value from a uniform distribution that is 1 unit wide. Observed rates that were reported to be exactly 0 are represented by soft uniform distributions with rates between [0, 1]. The soft intervals width selection is a conservative, arbitrary estimate on the basis of the recorded national average rates for HFMD (3.69 in 2008 and 8.68 in 2009, based on the corresponding sizes of the population of China and the reported HFMD cases).

For initial conditions, the initial spatial spread of infecteds, 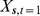, is given by the observed rates at 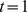. We start with no recovered individuals at *t* = 1. By considering an approximate disease duration of 1 week, the remaining part of the population are susceptibles to the disease. For the present illustration, we also assume the following:

(i) Relocation occurs sparsely during the 20-week study period, and it is accounted for by means of a Gaussian kernel function 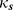 of bandwidth 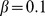 that results in factor sizes 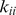 with a mean value 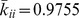 and a very skewed distribution towards high values (sample skewness −2.68). This means that on average, 97.55% of the population does not relocate during the study period.

(ii) Constant recovery 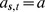 and transmission 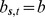 probabilities with initial values 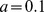 and 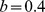, and variances 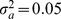 and 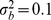.

The covariances 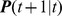 at the subsequent instances are based on the initial covariance that is computed for the initial spatial distribution of 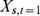. The covariance at *t* = 1 was estimated from the observed values at that instance, and was fitted by a correlation model with a nugget effect equal to 0.07 (rate variance units) and a spherical model with sill 0.07 (rate variance units) and spatial range 3°.

**Figure 10:**
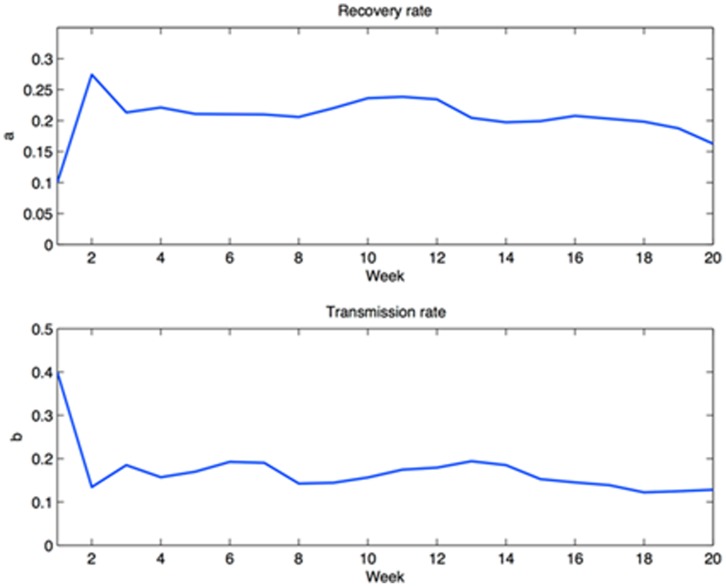
BME-SIR estimated transmission and recovery rates in the HFMD study.

On the basis of the above input, the BME-SIR method produces space-time distributions of the infected 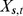, susceptible 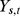, and recovered 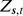, population fractions of HFMD throughout the 20-week study period. At each consecutive time instance, the general knowledge (BME-SIR model) drives the model parameters *a* and *b* progressively closer to the values that best interpret the present HFMD dataset. This process is also guided by updating the model with new specificatory (data) information at every time step. Figure 10 illustrates how the predicted parameter values from the current HFMD data reach equilibrium. The BME-SIR model predicts an approximate mean transmission rate 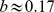, and an approximate mean recovery rate 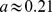. One observes that despite the arbitrary initial values of *a* and *b*, relatively accurate parameter estimates are reached relatively fast within about 2–4 weeks. For the scope of the present illustration, the above initial values have been selected rather arbitrarily. In more elaborate examples, it might be desirable to provide better-informed initial estimates for these rates. In the absence of expert knowledge, one possible way to tackle such cases could be to use existing data to obtain SIR-based regression estimates for the initial values of *a* and *b* [24].

**Figure 11:**
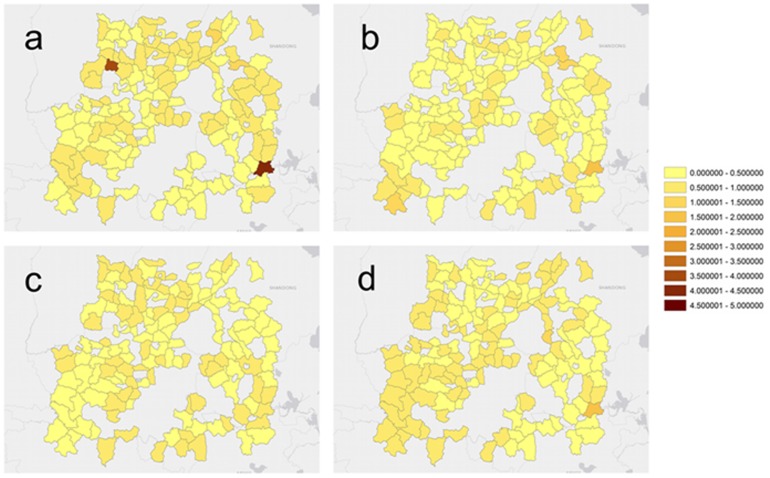
BME-SIR predicted population HFMD rates (cases per 10,000 people) in the study region for 4 selected week instances: (a) *t* = 5, (b) *t* = 10, (c) *t* = 15, and (d) *t* = 20.

**Figure 12:**
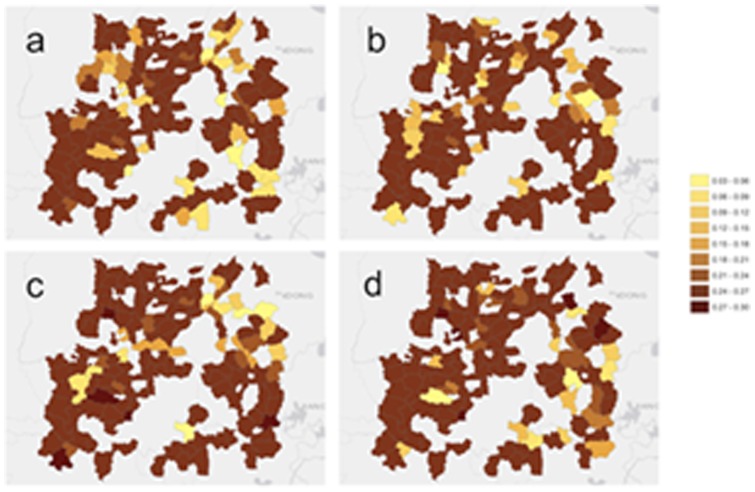
BME-SIR standard error for the predicted HFMD rates shown in **Fig. 11**.

Also, maps of the BME-SIR predicted mean of the infected distributions *X* ***_s_***_,*t*_ are produced for each of the 20 weeks of this study. Figure 11 shows these means inside the region of interest at selected week instances, and Fig. 12 illustrates the corresponding prediction error for those instances. The prediction error throughout the study was found to range between 0.0067 and 0.2884. These values are comparable to the corresponding predicted values, and reflect that the BME-SIR predictions also account for the uncertainty in the HFMD observations. In summary, this real-world case study indicates that BME-SIR can provide an informative overview of the disease evolution. Also, this application shows how BME-SIR can be effectively used to estimate the disease spread based on highly uncertain data, without any distributional assumptions. The BME-SIR estimation can assimilate both theoretical disease diffusion dynamics and the uncertain disease space-time data. As a result, the characteristics of disease evolution can be revealed over time, even in cases when the disease data are highly uncertain.

## Discussion

Characterizing space-time diffusion dynamics is a challenging effort due to complexities in population movement, disease transmission and recovery mechanisms, and uncertainties in observations. SIR models have for a long time been applied to study population-based disease diffusion at a specific site over time. To account for spatial diffusion, studies have been focusing on integrating detailed geographical information into SIR models by using multiple patches or networks to characterize the population movements and interactions. The detailed geographical topology can possibly consider the spatial heterogeneity of disease transmission. Under the framework of SIR-extended models, space-time disease diffusion can be studied based on the knowledge of the parameters of disease dynamics inferred from data; e.g. transmission rate. However, in most cases, detailed and accurate information on population interactions is partially available. In addition, infectious disease data can be sparse and highly noisy and, therefore, the characteristics of disease dynamics are highly uncertain, especially at the initial stage of the disease outbreak. In this study, we account for uncertainties in the available data, as well as the unknown characteristics of disease dynamics, by proposing a spatiotemporal BME-SIR method of infectious disease spread. Based upon the SIR concept, the BME framework allows space-time disease modeling to account for patch-based population movements and multiple-sourced uncertainties, including: 1) unknown prior knowledge of disease dynamics, i.e. transmission and recovery rates, and 2) uncertainties in disease data from direct or indirect observations.

**Figure 13:**
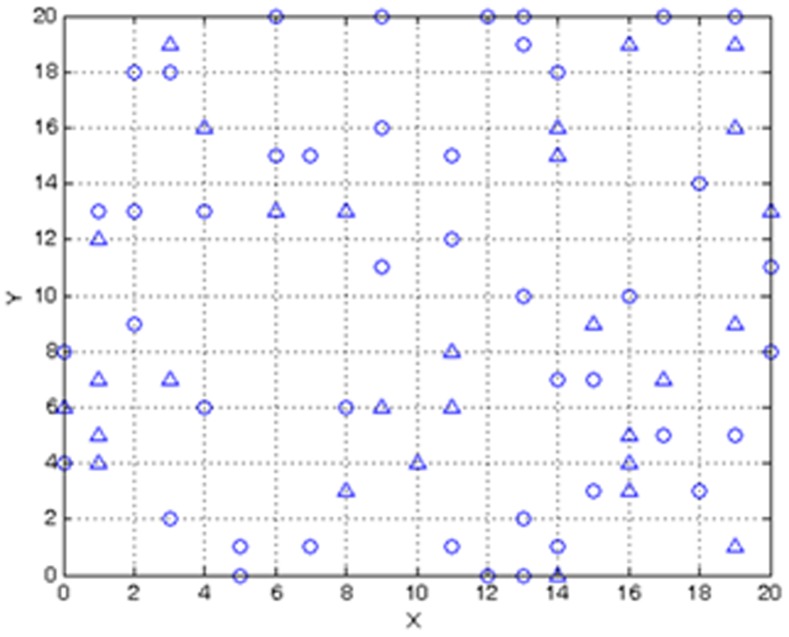
Spatial distribution of sampling locations (data serving as input to BME-SIR). Circles: hard (accurate) data. Triangles: soft (uncertain) data.

**Figure 14:**
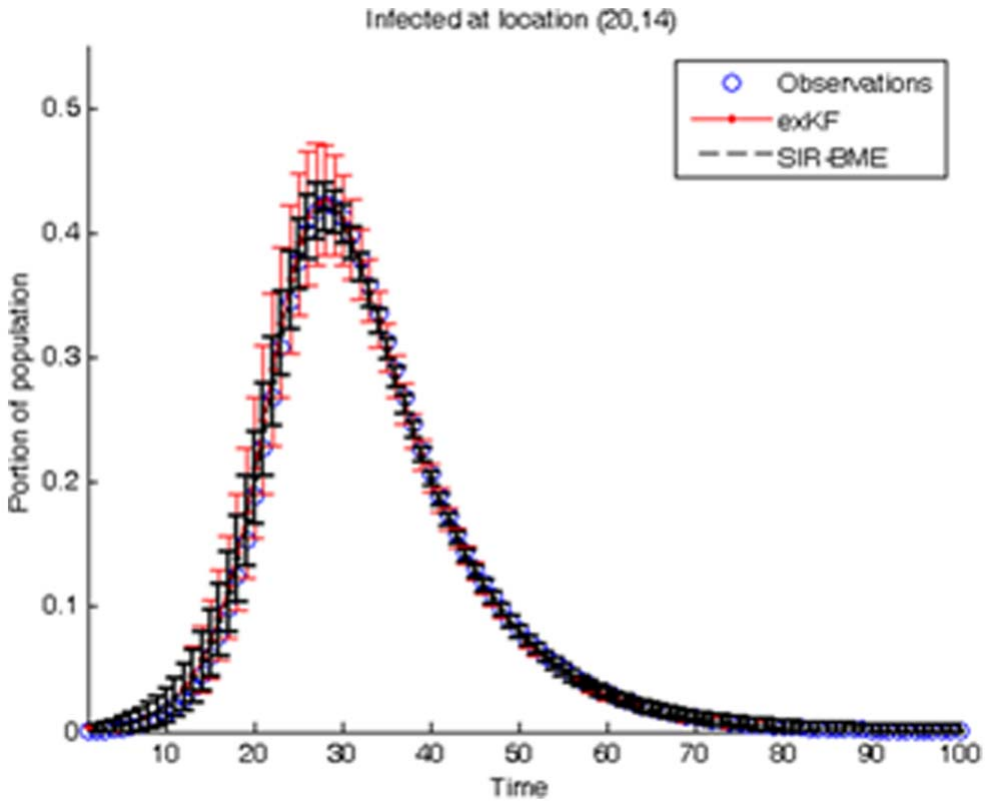
Comparison between the simulated infected population fractions at location 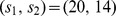
,at different times *t*, and the corresponding exKF and BME-SIR estimates.

**Figure 15:**
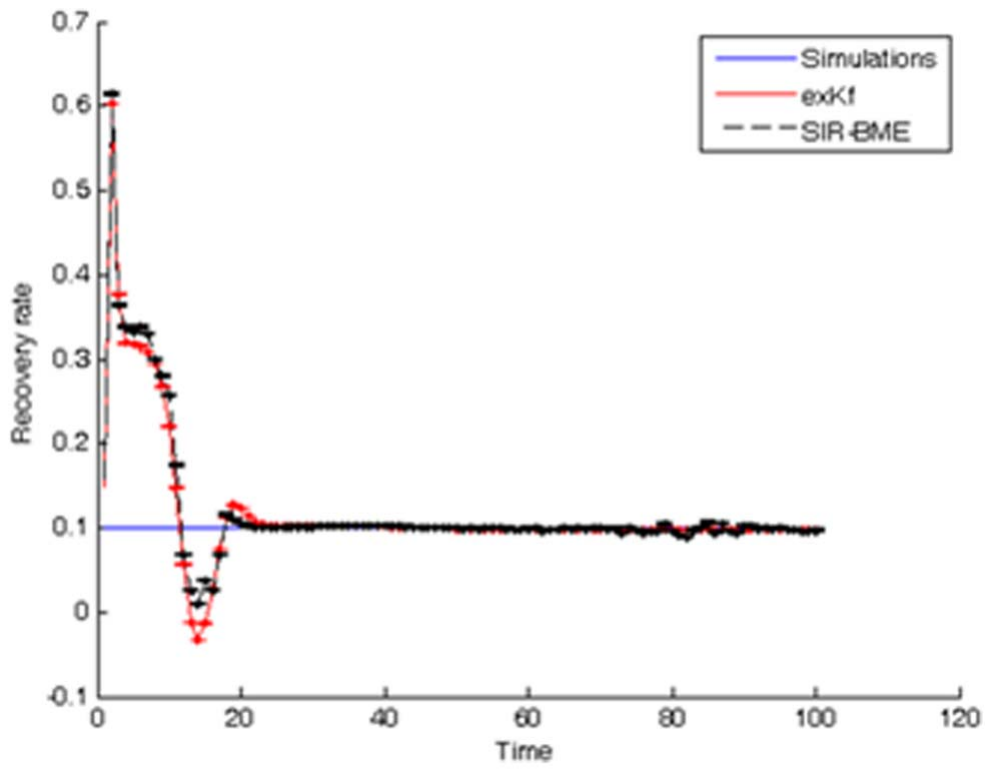
Comparison between the simulated and estimated recovery rates using the exKF and BME-SIR methods.

To gain additional insight of the complete space-time SIR model, we progressively simplified the model to: (*a*) be expressed in a linear state-space form (i.e., using the *φ*-function approximation), and (*b*) be described by analytical solutions (i.e., static population assumption). Overall, the linearized SIR model showed good performance in reproducing the infected, susceptible and recovered population fractions, their empirical correlations (Fig. 4), and in inferring the transmission and recovery rates from data with low estimation error (see Fig. 13 and the error bars in Figures 14–15) and minimal computational effort. This makes the developed BME-SIR model an ideal framework for real-world application studies, where one needs to model the spread of infectious diseases in space-time, for different initial conditions, using a minimum number of parameters. The latter should suffice to reproduce the covariance structure of the susceptible, infected and recovered population fractions at different times over the whole simulation grid. The BME-SIR model effectively achieves this goal using only two model parameters (transmission and recovery rates), which can be easily inferred from data and, in more demanding studies, can vary systematically in both space and time.

For the purposes of disease control, real-time prediction of space-time disease spread is required by governmental agencies. For the cases of emerging infectious diseases, real-time prediction is essential due to higher risks and increased uncertainties in the infected cases and disease parameters, e.g. reproduction number. Modeling of the spatiotemporal patterns of emerging disease spread involves uncertainties from various sources, e.g. model uncertainty, parameter and data uncertainties. Data assimilation approaches can continuously incorporate new observations into the physical process, and has been widely used in a variety of applications, e.g. geosciences [25], [26]; however, relatively few studies investigated the application of data assimilation approaches to infectious disease predictions [27]. Kalman filter is one of the most widely used data assimilation approaches for real-time prediction. It is based upon the state-space model and assumes the model and observation uncertainties are Gaussian-distributed. The BME-SIR method combines the linearized state-space model (i.e. general knowledge), with disease data with various levels of uncertainty (i.e. site-specific knowledge), to produce real-time disease estimates. Similar to the other data assimilation methods (e.g. Kalman filter), BME-SIR can update the model predictions, whenever new observations become available. The proposed spatiotemporal BME-SIR filtering framework can incorporate multi-sourced uncertainties (like exKF), and produce real-time disease estimates in a space-time domain. The distinction between exKF and BME-SIR methods is that BME-SIR can account for data uncertainties without any underlying distributional assumptions. Real-time estimates of infected population fractions as well as the transmission and recovery rates are shown in Figs. 14 and 15. Note that the estimates obtained by BME-SIR and exKF are consistently updated as new observations become available. The parameter values predicted by BME-SIR and exKF reach an equilibrium after about 10 weeks (see Fig. 10). Spatial maps of the predicted mean of the infected population fraction, 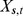, were produced on a spatial grid of 30×25 = 750 nodes for each of the 20 weeks of the study; see Fig. 11.

Various extensions of this work are under development. Among others, the definition of a continuous-time version of the model consistent with the discrete-time formulation studied here. Also, the consideration of heterogeneous propagation of infection through non-homogeneous kernels associated with spatial spread; for instance, in terms of spatial deformation accounting for covariate effects.

## Methods

### The Space-Time Disease Model

Disease spread is a fundamentally spatiotemporal phenomenon, the rigorous study of which should account for a number of uncertainty sources (e.g. disease variability, imperfect observation conditions, population density fluctuations, physiographic features, meteorological matters). This constitutes sufficient motivation for extending the original SIR model in the space-time context under conditions of real world uncertainty. The distribution of the fraction of infected population is represented as a *spatiotemporal random field* 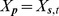 [28], [29], where 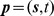 denotes a physical location with spatial coordinates 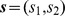 at time *t*. Similarly, 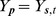 and 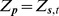 are random fields representing the distributions, respectively, of the fraction of the population that is susceptible to become infected and the fraction of the population that has recovered and is immune. The basic relationships between 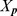, 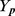, 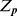 are, 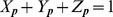, 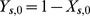 and 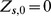, where 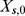, 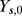, and 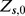 denote the initial conditions (IC) of the corresponding population fractions [30]. The proposed modeling of the combined space-time distributions 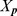, 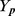, 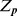 is described by the following generalized SIR model in continuous time (for the discrete time case, see [19] and references therein),
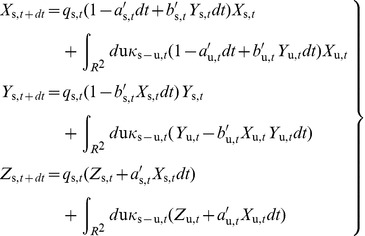
(1a-c)where 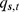 is the population fraction that resides (i.e., does not displace from) at the space-time domain 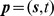, 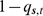 is the fraction that migrates during the time period 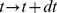, 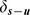 is the delta function, and 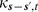 is a spatially homogeneous kernel (e.g., Gaussian kernel with finite variance) that controls population movement across space, with spatial integral being equal to 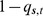. In addition, 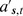 is the rate [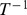] that an infected individual, at the space-time domain 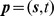, recovers and becomes immune, and 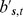 is the corresponding rate [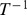] of infection transmission during an encounter of one infected and one susceptible individual. Note that 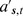 and 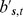 allow one to include information about regional topography and local climatic conditions. Moreover, under conditions of in situ disease control (quarantine, vaccination etc.), transmission and recovery rates are time-varying. The stochastic SIR model (1a–c) is, by construction, a composite space-time representation of disease spread. The space-time covariances of 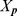, 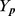, 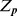 are derived from the SIR model (details in File S1).

In the case when 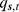, 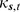, 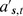 and 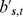 vary slowly with *t* (say 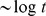), or they are constant in time (i.e. 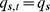, 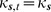, 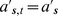 and 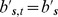), Eqs (1a–c) satisfy the following set of integrodifferential space-time SIR equations:
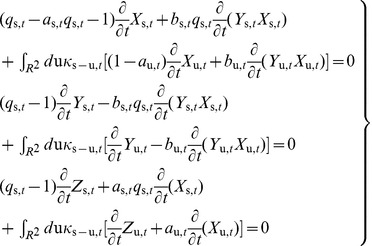
(2a-c)where 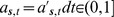 is the probability that an infected individual, at 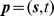, recovers and becomes immune, and 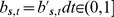 is the probability of infection transmission during an encounter of one infected and one susceptible individual.

BME is a stochastic approach for spatiotemporal modeling and prediction in conditions of space-time heterogeneity and in-situ uncertainty [31]. BME disease modeling can rigorously integrate different disease knowledge bases, e.g. laws of disease evolution dynamics with available space-time disease datasets to provide informative and accurate predictions of disease spread. BME distinguishes between two major disease knowledge bases (KBs): (a) the core or general KB, *G*-KB, which includes physical and biological laws (e.g., the SIR model); and (b) the site-specific KB, *S*-KB, which includes hard or exact data and soft or uncertain data (e.g., observations of disease counts across space-time as exact numerical values or as interval and probability distributions of possible values). The BME method integrates both knowledge bases (i.e. 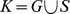) in terms of the following fundamental BME equations [17], [18]

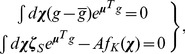
(3)where the vector 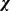 denotes realizations of the distribution of 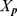 in space-time (clearly, the equation could be written in terms of 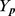 and 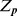 realizations, as well), 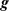 is a vector of functions that represent stochastically the *G*-KB under consideration (the bar denotes statistical expectation), and 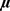 is a vector of coefficients that depend on the space-time coordinates (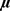 is linked to 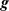). BME is not constrained by assumptions commonly used in the literature, such as Gaussian probability distribution of disease attributes and linear estimator forms. In the present case, disease evolution is governed by the space-time SIR model (1a–c); therefore, 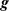 includes the mean, covariance and cross-covariance functions of 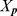, 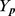, and 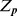 derived from the theoretical space-time SIR model (1a–c) to account for disease trends and correlation patterns in the population at different geographical locations and during different time periods. (More details about the space-time BME-SIR method will be given later.) 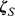 represents the available *S*-KB, which can be direct or indirect disease observations across space and time in terms of fixed values or probabilistic distributions of disease attributes. *A* is a normalization parameter, and 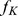 is the probability density function (pdf) of estimated disease counts at each space-time point (the subscript *K* means that 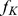 is based on the blending of core and site-specific KBs). 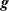 and 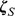 are the inputs to Eqs. (3a–c), whereas the unknowns 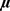 and 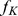 vary from place to place and from time to time. Estimates of the unknown parameters in vector 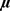 are generally obtained by means of optimization techniques [32]–[34]. In this study, since the means and covariances of the space-time SIR model are used in the G-KB, the unknown parameters in 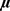 can be directly derived from analytical statistical physics formulas [17]. The estimation of 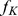 at different spatial locations and temporal instances (i.e. space-time points) is based on operational Bayesian theory which does not require any distributional assumptions [35].

Another way to look at Eqs (3) is that they generate a stochastic solution of the integodifferential SIR equations (2a–c) that –compared to the standard solutions of general integodifferential equations— has the unique feature to also account for several other kinds of available knowledge (hard and soft data, empirical relationships, secondary information) and multi-sourced uncertainties (in the composite space-time disease variation, the records of infected individuals etc.). In section IV, the BME-SIR disease dynamics model will be discussed together with certain simulation cases, in which the BME-SIR method fuses the SIR disease model (*G*-KB) and disease related observations and records (*S*-KB).

### Special Cases of SIR Dynamics with Closed-Form Solutions

Valuable insight about the spatiotemporal SIR dynamics is gained by considering some special cases of the SIR model (1a–c). For example, if the space-time dependence of the infected (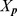) and the susceptible (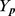) distributions satisfy certain separability conditions (which assure system linearity), a function 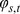 can be defined that has a smooth shape similar to that of the covariance function of 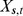 and 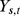 (details in File S1). For example, 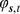 may be chosen to be a monotonically decreasing function of time *t* with sufficient flexibility to represent the behavior of the population fraction that is susceptible to infection; see also section VI below.

Let us start by assuming that during the time period of interest the population is static (i.e., it does not move in space) while the disease spreads (i.e., 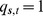), and the infecteds IC (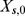) are spatially homogeneous. Note that in the case when 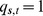, the time-independent kernel that controls population movement across space, 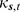, does not play any role and, hence, the integral terms in Eqs (1a–c) can be neglected. In this case, Eqs (1a–c) reduce to
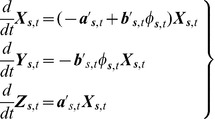
(4a-c)with ICs, 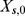, 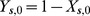, 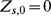. 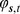 is a function with a smooth shape similar to that of the covariance function of 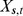 and 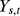; see above. In a sense, the SIR model in Eqs. (4a–c) is an extension, in a composite space-time domain, of the mainstream and purely temporal SIR model [5], [10], [12]. The closed-form solution of Eqs (4a–c) is
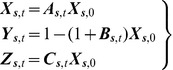
(5a-c)where, 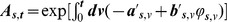, 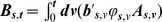, 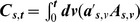, and 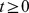.

The mathematical expressions of the covariance and cross-covariance functions of the disease variables 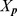, 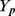 and 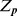 are shown in Table 1. One can see that all space-time disease covariance and cross-covariance functions: (*a*) depend on 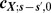 (covariance between the ICs 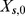 and 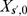); and (*b*) are broadly non-homogeneous (in space) and non-stationary (in time). In the case when 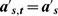, 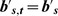,and 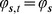 are constant in time, the parameters 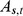, 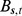, and 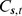 receive the following closed analytical forms
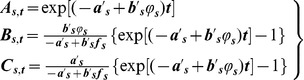
(6a-c)Below we consider some numerical applications of the SIR model presented above.

**Table 1:** Covariances and cross-covariances of 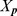, 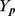 and 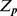.

	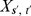	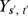	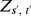
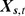	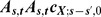	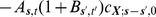	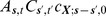
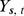	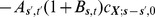	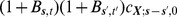	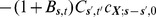
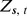	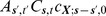	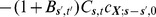	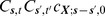

### SIR in the BME setting

In practice, spatiotemporal disease modeling is performed in uncertain conditions, e.g., erratic disease observations, incomplete prior knowledge of disease transmission and recovery rates [36]. In most cases of SIR modeling, a rather incomplete knowledge of the in situ susceptible and recovered population fractions is possible. The numerical study shows that when the disease observations are uncertain and follow a non-Gaussian law, the BME method in Eq. (3)([28], [32], [37], [38]) can further improve updating in the SIR model by representing erratic observations in the form of probabilistic data and by incorporating transmission and recovery rate uncertainties. In the BME framework, the core or general KB (*G*) includes the SIR equations and the associated disease covariance models, whereas the site-specific KB (*S*) includes the uncertain infected observations and the initial conditions of the transmission and recovery rates. The SIR states are then formulated in the matrix form
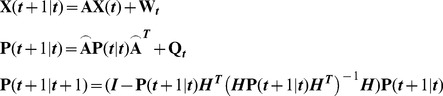
(8)where 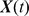 and 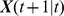 are vectors containing the current and predicted states of infected disease counts and the rates of the SIR model; i.e. recovery rate 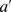 and transmission rate 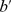, respectively. 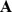 and 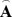 are the transition and Jacobian matrices characterizing the dynamics of the SIR model. 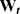 models the uncertainty of infected states across space, which cannot be represented by SIR modeling, and is characterized by the covariance matrix 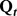. The observation matrix 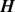 contains only zeros and ones indicating data presence across space. 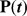, 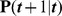 and 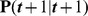 are the current, predicted and updated state covariances. Equation (8) involve the general KB containing the stochastic properties of disease dynamics (details of the matrix formulation of Equation (S8) shown in File S1). Concerning the site-specific KB, for estimation purposes the hard (accurate) data are randomly sampled over time at 44 spatial cells of the disease grid mentioned earlier. In addition, soft (uncertain) data that follow uniform probability distributions with uncertain ranges are sampled from another set of 29 cells. The sample locations are shown in Fig. 13.

**Figure 16:**
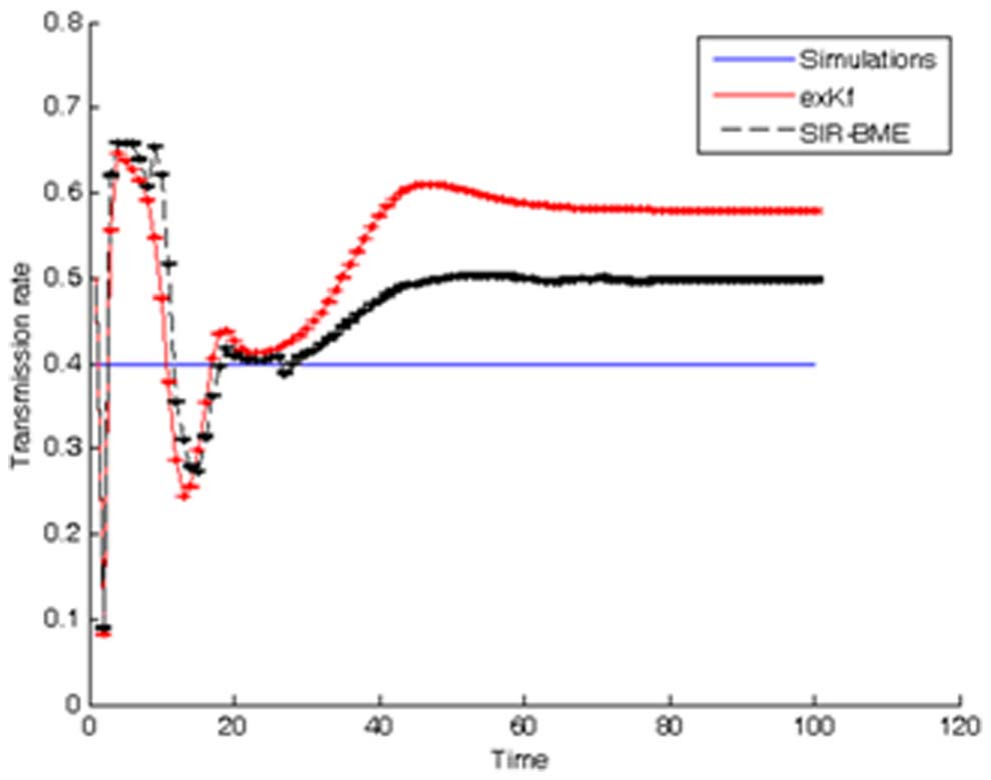
Comparison between simulated and estimated transmission rates using the exKF and BME-SIR methods.

Numerical comparisons between the simulation results for model prediction and parameter estimation by the BME-SIR method are shown in Figs. 14, 15, 16. For comparison purposes, the results obtained using the extended Kalman filter (exKF) are also shown (technical details of the exKF SIR model can be found in [19]). Figure 14 shows that both methods predict almost equally well the infected population fractions at different times *t* (mean-square errors: 23.93 for BME-SIR and 28.92 for the exKF). Improvements in estimation uncertainty gained by using the BME-SIR over the exKF method are also shown. Similar results were obtained for the susceptible and recovered population fractions. Figs. 15, 16 demonstrate the performance of the two methods in estimating the transmission and recovery rates at different times *t*. One sees that both methods provide effective estimates of the SIR recovery rates at all times, but the corresponding estimates of the transmission rates for large times *t* (i.e., *t*>50) are poor. The changes in the recovery and transmission rates show some interesting temporal patterns. When *t* is small (e.g., *t*<10), the estimated rates are closely associated with their initial guess and therefore the deviations of both the BME-SIR and exKF rate estimates are large. The improvement of rate estimation is shown over time. The transmission rate estimation accuracy obtained by both methods is low when *t*>40. This is due to the low portion of susceptibles after time *t* = 40 (i.e., the percentage of susceptible population after *t*>40 is less than 3%), which yields transmission rate estimates insensitive to observations. However, even in the case of low infected population fractions, both the BME-SIR and exKF methods produce accurate recovery rate estimates. Clearly, the SIR model is more sensitive to changes in the recovery rate rather than the transmission rate (Figs. 15–16). As a result, real-time data assimilation should lead to better estimates of real-time transmission rates.

## Supporting Information

File S1 **Supplementary materials.**
(DOC)

## References

[1] Eggo RM, Cauchemez S, Ferguson NM (2011) Spatial dynamics of the 1918 influenza pandemic in England, Wales and the United States. Journal of the Royal Society Interface 8: 233–243. 10.1098/rsif.2010.0216 PMC3033019 20573630

[2] Grenfell BT, Bjornstad ON, Finkenstadt BF (2002) Dynamics of measles epidemics: Scaling noise, determinism, and predictability with the TSIR model. Ecological Monographs 72: 185–202.

[3] Grenfell BT, Bjornstad ON, Kappey J (2001) Travelling waves and spatial hierarchies in measles epidemics. Nature 414: 716–723. 11742391 10.1038/414716a

[4] Cummings DAT, Iamsirithaworn S, Lessler JT, McDermott A, Prasanthong R, et al (2009) The Impact of Changes in Human Demography on Cycles of Dengue Hemorrhagic Fever Incidence in Thailand. American Journal of Epidemiology 169: S40–S40.

[5] Anderson RM, May RM, Boily MC, Garnett GP, Rowley JT (1991) The Spread of Hiv-1 in Africa - Sexual Contact Patterns and the Predicted Demographic-Impact of Aids. Nature 352: 581–589. 1865922 10.1038/352581a0

[6] Riley S (2007) Large-scale spatial-transmission models of infectious disease. Science 316: 1298–1301. 17540894 10.1126/science.1134695

[7] Riley S, Ferguson NM (2006) Smallpox transmission and control: Spatial dynamics in Great Britain. Proceedings of the National Academy of Sciences of the United States of America 103: 12637–12642. 16894173 10.1073/pnas.0510873103 PMC1567931

[8] Ferguson NM, Keeling MJ, Edmunds WJ, Gant R, Grenfell BT, et al (2003) Planning for smallpox outbreaks. Nature 425: 681–685. 14562094 10.1038/nature02007 PMC7095314

[9] Schrödle B, Held L, Rue H (2012) Assessing the Impact of a Movement Network on the Spatiotemporal Spread of Infectious Diseases. Biometrics 68: 736–744. 22171626 10.1111/j.1541-0420.2011.01717.x

[10] Allen LJS, Burgin AM (2000) Comparison of deterministic and stochastic SIS and SIR models in discrete time. Mathematical Biosciences 163: 1–33. 10652843 10.1016/s0025-5564(99)00047-4

[11] Alonso D, McKane AJ, Pascual M (2007) Stochastic amplification in epidemics. Journal of the Royal Society Interface 4: 575–582. 10.1098/rsif.2006.0192 PMC2373404 17251128

[12] West RW, Thompson JR (1997) Models for the simple epidemic. Mathematical Biosciences 141: 29–39. 9077078 10.1016/s0025-5564(96)00169-1

[13] Anderson RM, May RM, Ibrahim MA (1991) Infectious diseases of humans: dynamics and control. Oxford; New York: Oxford University Press. viii: , 757 p. p.

[14] Lloyd AL, Jansen VAA (2004) Spatiotemporal dynamics of epidemics: synchrony in metapopulation models. Mathematical Biosciences 188: 1–16. 10.1016/j.mbs.2003.09.003 14766090

[15] Keeling MJ, Gilligan CA (2000) Metapopulation dynamics of bubonic plague. Nature 407: 903–906. 11057668 10.1038/35038073

[16] Keeling MJ, Eames KTD (2005) Networks and epidemic models. Journal of the Royal Society Interface 2: 295–307. 10.1098/rsif.2005.0051 PMC1578276 16849187

[17] Christakos G (2000) Modern Spatiotemporal Geostatistics. New York: Oxford University Press.

[18] Christakos G, Olea R, Serre M, Yu H, Wang L (2005) Interdisciplinary Public Health Reasoning and Epidemic Modelling: The Case of Black Death: New York, N.Y: Springer-Verlag.

[19] Angulo J, Yu H-L, Langousis A, Madrid AE, Christakos G (2012) Modeling of space-time infectious disease spread under conditions of uncertainty. International Journal of Geographical Information Science Available online

[20] Aitchison J (1986) The statistical analysis of compositional data. London; New York: Chapman and Hall. xv: , 416 p. p.

[21] Pawlowsky-Glahn V, Buccianti A (2011) Compositional data analysis: theory and applications. Chicester, West Sussex; Hoboken, N.J.: Wiley. xxi: , 378 p.

[22] Li L (2010) Review of hand, foot and mouth disease. Frontiers of Medicine in China 4: 139–146.

[23] Wang JF, Guo YS, Christakos G, Yang WZ, Liao YL, et al (2011) Hand, foot and mouth disease: spatiotemporal transmission and climate. International Journal of Health Geographics 10. 10.1186/1476-072X-10-25 PMC3079592 21466689

[24] Wang JF, McMichael AJ, Meng B, Becker NG, Han WG, et al (2006) Spatial dynamics of an epidemic of severe acute respiratory syndrome in an urban area. Bulletin of the World Health Organization 84: 965–968. 17242832 10.2471/blt.06.030247 PMC2627565

[25] Kalnay E (2003) Atmospheric modeling, data assimilation, and predictability. Cambridge, U.K.; New York: Cambridge University Press. xxii: , 341 p., 344 p. of plates p.

[26] Park SK, Xu L (2009) Data assimilation for atmospheric, oceanic and hydrologic applications. Heidelberg: Springer,.

[27] Bettencourt LA, Ribeiro R, Chowell G, Lant T, Castillo-Chavez C (2007) Towards Real Time Epidemiology: Data Assimilation, Modeling and Anomaly Detection of Health Surveillance Data Streams. In: Zeng D, Gotham I, Komatsu K, Lynch C, Thurmond M, et al.., editors. Intelligence and Security Informatics: Biosurveillance: Springer Berlin Heidelberg. pp. 79–90.

[28] Christakos G, Hristopulos DT (1998) Spatiotemporal Environmental Health Modelling: A Tractatus Stochasticus. Boston, MA,: Kluwer Academic Publishers.

[29] Rohani P, Earn DJD, Grenfell BT (1999) Opposite patterns of synchrony in sympatric disease metapopulations. Science 286: 968–971. 10542154 10.1126/science.286.5441.968

[30] Ball F, Neal P (2002) A general model for stochastic SIR epidemics with two levels of mixing. Mathematical Biosciences 180: 73–102. 12387917 10.1016/s0025-5564(02)00125-6

[31] Christakos G (1990) A Bayesian/maximum-entropy view to the spatial estimation problem. Mathematical Geology 22: 763–776.

[32] Yu HL, Kolovos A, Christakos G, Chen JC, Warmerdam S, et al (2007) Interactive spatiotemporal modelling of health systems: the SEKS-GUI framework. Stochastic Environmental Research and Risk Assessment 21: 555–572.

[33] Baker R, Christakos G (2007) Revisiting prior distributions, Part II: Implications of the physical prior in maximum entropy analysis. Stochastic Environmental Research and Risk Assessment 21: 435–446.

[34] Dudik M, Phillips SJ, Schapire RE (2007) Maximum entropy density estimation with generalized regularization and an application to species distribution modeling. Journal of Machine Learning Research 8: 1217–1260.

[35] Christakos G (2002) On the assimilation of uncertain physical knowledge bases: Bayesian and non-Bayesian techniques. Advances in Water Resources 25: 1257–1274.

[36] Elderd BD, Dukic VM, Dwyer G (2006) Uncertainty in predictions of disease spread and public health responses to bioterrorism and emerging diseases. Proceedings of the National Academy of Sciences of the United States of America 103: 15693–15697. 17030819 10.1073/pnas.0600816103 PMC1592533

[37] Orton TG, Lark RM (2007) Accounting for the uncertainty in the local mean in spatial prediction by Bayesian Maximum Entropy. Stochastic Environmental Research and Risk Assessment 21: 773–784.

[38] Christakos G, Olea RA (2005) New space-time perspectives on the propagation characteristics of the Black Death epidemic and its relation to bubonic plague. Stochastic Environmental Research and Risk Assessment 19: 307–314.

