# Correction: Spatiotemporal Infectious Disease Modeling: A BME-SIR Approach

**DOI:** 10.1371/annotation/4adf8407-a5b8-4f4b-877d-e8b944f0e6ee

**Published:** 2013-10-03

**Authors:** Jose Angulo, Hwa-Lung Yu, Andreas Langousis, Alexander Kolovos, Jinfeng Wang, Ana Esther Madrid, George Christakos

There was an error in the name of the second author. The correct name of this author is: Andreas Langousis. 

