# Supplementary material for: Spatiotemporal Infectious Disease Modeling: A BME-SIR Approach
Source: PLoS One. 2013 Sep 27;8(9):e72168. doi: 10.1371/journal.pone.0072168 (PMC3785461; doi:10.1371/journal.pone.0072168)
Supplement: File S1 — Supplementary materials. (DOC) [file pone.0072168.s001.doc]

**Supplementary materials**

*Introducing the $\phi$-function for SIR model linearization*

The space-time covariances of $X_{s, t}$, $Y_{s, t}$ and $Z_{s, t}$ can be derived from the SIR Eqs. (1a-c). For the purposes of demonstration, the space-time covariance of the infected population fraction $X_{p}$ can be expressed as

(S1)

where$c_{X; s, t, s', t'}=\mathrm{cov}(X_{s, t}, X_{s', t'})$. Valuable insight can be still gained by considering some special cases of the SIR model in Eqs (1a-c). For example, if the space-time dependence of the incidence ($X_{p}$) and the susceptible ($Y_{p}$) fields satisfy the relationship

$\mathrm{cov}(X_{s, t}Y_{s, t}, X_{s', t'}Y_{s', t'})=\mathrm{cov}(X_{s, t}, Y_{s', t'}X_{s', t'})\mathrm{cov}(X_{s', t'}, Y_{s, t}X_{s, t}) c_{X; s, t, s', t'}^{-1}$ , (S2)

a function $\phi_{s, t}$ can be defined such that

. (S3a-c)

The $\phi$-function has a smooth shape similar to that of the covariance function of $X_{s, t}$ and $Y_{s, t}$. For example, $\phi_{s, t}$ may be chosen to be a monotonically decreasing function of time $t$ with sufficient flexibility to represent the behavior of the population fraction that is susceptible to infection. Using the $\phi$-function assumption, the composite space-time SIR model (1a-c) reduces to

(S4a-c)

This simplification leads to linearization of the model with respect to the stochastic components. The space-time covariances of the reduced SIR model in Eqs. (S4a-c), can be simplified from Eq. (S1) and written as

(S5)

The covariances and cross-covariances among $X_{p}$, $Y_{p}$, and $Z_{p}$ can be derived accordingly; see .

*Formulation of SIR dynamics for state-space modeling*

The state-space model considers spatiotemporal disease dynamics as a first-order differential equation of the states that are physically meaningful, but may not be observable. The state-space model of Eq. (8) in this study can be written as

$\left[\begin{matrix} X_{1, t+1} \\ X_{2, t+1} \\ \vdots\\ X_{n, t+1} \\ a_{t+1} \\ b_{t+1} \end{matrix}\right]=\mathbf{A}\left[\begin{matrix} X_{1, t} \\ X_{2, t} \\ \vdots\\ X_{n, t} \\ a_{t} \\ b_{t} \end{matrix}\right]+\left[\begin{matrix} w_{1, t} \\ w_{2, t} \\ \vdots\\ w_{n, t} \\ w_{a.t} \\ w_{b.t} \end{matrix}\right]$ (S6)

where $a_{t}$ and $b_{t}$ are the recovery and transmission rates respectively, and

$\mathbf{A}=\left[\begin{matrix} q_{1}(1-a_{t}+b_{t}\phi_{1,t}) & k_{21}(1-a_{t}+b_{t}\phi_{2,t}) & \cdots& k_{n1}(1-a_{t}+b_{t}\phi_{n,t}) & 0 & 0 \\ k_{12}(1-a_{t}+b_{t}\phi_{1,t}) & q_{2}(1-a_{t}+b_{t}\phi_{2,t}) & \cdots& k_{n2}(1-a_{t}+b_{t}\phi_{n,t}) & 0 & 0 \\ \vdots& \vdots& \ddots& \vdots& \vdots& \vdots\\ k_{1n}(1-a_{t}+b_{t}\phi_{1,t}) & k_{2n}(1-a_{t}+b_{t}\phi_{2,t}) & \cdots& q_{n}(1-a_{t}+b_{t}\phi_{n,t}) & 0 & 0 \\ 0 & 0 & 0 & 0 & 1 & 0 \\ 0 & 0 & 0 & 0 & 0 & 1 \end{matrix}\right]$ (S7)

(S8)

where $\tilde{\varphi}_{i, t}=(Y_{i,t}^{2}+Y_{i, t}Z_{i, t})(X_{i, t}+Y_{i, t}+Z_{i, t})^{-2}$; see [1].

*Availability of Hand-Foot-Mouth Disease Dataset*

The dataset used in this study was obtained from China Disease Center of Disease Control. Data can be accessed upon requested by contacting Dr. Jinfeng Wang with email wangjf@lreis.ac.cn

Reference

1. Angulo J, Yu H-L, Langousis A, Madrid AE, Christakos G (2012) Modeling of space-time infectious disease spread under conditions of uncertainty. International Journal of Geographical Information Science. DOI:10.1080/13658816.2011.648642
